# Is there a causal association between gestational diabetes mellitus and immune mediators? A bidirectional Mendelian randomization analysis

**DOI:** 10.3389/fendo.2024.1358144

**Published:** 2024-04-19

**Authors:** Zhangxin Ji, Chenxu Zhang, Jingjing Yuan, Qing He, Xinyu Zhang, Dongmei Yang, Na Xu, Jun Chu

**Affiliations:** ^1^ Key Laboratory of Xin’an Medicine, Ministry of Education, Anhui University of Chinese Medicine, Hefei, Anhui, China; ^2^ School of Graduate, Anhui University of Chinese Medicine, Hefei, Anhui, China; ^3^ Research and Technology Center, Anhui University of Chinese Medicine, Hefei, Anhui, China; ^4^ State Key Laboratory of Tea Plant Biology and Utilization, School of Tea and Food Science and International Joint Laboratory on Tea Chemistry and Health Effects of Ministry of Education, Anhui Agricultural University, Hefei, Anhui, China; ^5^ Institute of Surgery, Anhui Academy of Chinese Medicine, Anhui University of Chinese Medicine, Hefei, Anhui, China

**Keywords:** gestational diabetes mellitus, immunity, causal inference, genetic variation, Mendelian randomization

## Abstract

**Background:**

Diabetes that only appears or is diagnosed during pregnancy is referred to as gestational diabetes mellitus (GDM). The maternal physiological immune profile is essential for a positive pregnancy outcome. However, the causal relationship between GDM and immunophenotypes is not fully defined.

**Methods:**

Based on the high-density genetic variation data at the genome-wide level, we evaluated the logical associations between 731 specific immune mediators and GDM using bidirectional Mendelian randomization (MR). The inverse variance weighted (IVW) was the main method employed for MR analysis. We performed multiple methods to verify the robustness and dependability of the MR results, and sensitivity measures were applied to rule out potential heterogeneity and horizontal pleiotropy.

**Results:**

A substantial causal association between several immune mediators and GDM was detected. After FDR testing, *HLA DR++ monocyte %leukocyte* and *HLA DR on plasmacytoid DC* were shown to increase the risk of GDM; in contrast, *CD127 on CD28+ CD45RA+ CD8br* and *CD19 on PB/PC* were shown to attenuate the effect of GDM. Moreover, the progression of GDM has been shown to decrease the maternal levels of *CD39+ activated Treg AC*, *CD39+ activated Treg %CD4 Treg*, *CD39+ resting Treg AC*, *CD39+ resting Treg %CD4 Treg*, and *CD39+ CD8BR %T cell*.

**Conclusions:**

Our findings support a possible causal association between GDM and various immunophenotypes, thus facilitating the provision of multiple options for preventive recognition as well as for the diagnostic and therapeutic management of GDM in clinical practice.

## Introduction

Numerous physiological changes take place in a woman’s body during pregnancy. Throughout the long gestation period, the main energy pathway received by the fetus is glucose from the mother’s placenta (1). While pregnancy progresses, the fetal need for glucose grows, which leads to gestational diabetes mellitus (GDM) in women with otherwise healthy glucose metabolism or potentially impaired glucose tolerance prior to pregnancy (2, 3). As a common complication of pregnancy worldwide, uncontrolled GDM poses a serious threat to the mother, fetus, and newborn, increasing the likelihood of adverse pregnancy reactions (e.g., gestational hypertension, infection, and metabolic ketoacidosis) as well as malignant pregnancy outcomes (e.g., preterm abortion, neonatal hypoglycemia, and postpartum depression) (4). The focus of GDM prevention efforts is on preconception or early pregnancy, with only a minority of women with GDM requiring pharmacological treatment, and the identification of reliable underlying risk markers is valuable for the timely detection and prognosis of GDM (5).

The growth of a fetus from conception to successful delivery is a significant challenge for the physiological functions and regulatory systems of the mother (6). Patients with GDM frequently experience metabolic issues, such as increased insulin resistance, and generally suffer from systemic mild inflammation and immune dysregulation (7). In GDM, various types of immune cells, particularly regulatory T cells (Tregs), adapt spontaneously to prevent pregnancy interruption. Besides this, there is a proportional increase in circulating monocyte activation and an elevated level of cytokines, including IL-12 and IL-23, in mid to late gestation compared to non-pregnant women (8). It is quite predictable that changes in the quantity or function of immune mediators are involved in the development of GDM. Regrettably, the conclusions of the current studies on the correlation between maternal immune profiles and GDM are not entirely consistent, which may be due to factors such as differences in the samples and flaws in the design.

**Figure 1:**
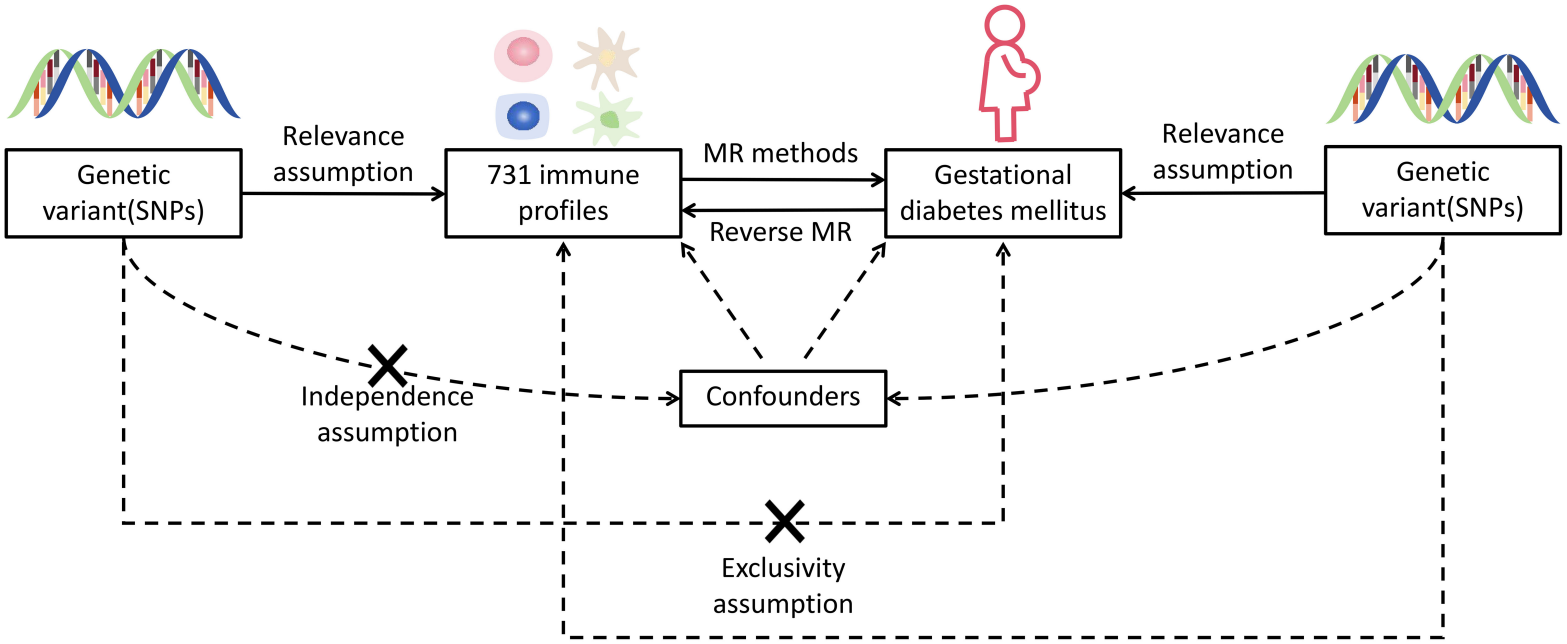
Hypothetical design of MR studies. Relevance: there is a significant relationship between exposure variables and genetic variations: genetic variants and confounding factors are independent of each other; exclusivity: genetic variation affects outcomes only through exposure and not by other means. X means no direct association.

Nowadays, genome-wide association study (GWAS) and Mendelian randomization (MR) make it feasible to estimate the causal associations between immune traits and disease events on a large scale. GWAS identifies genome-wide sequence variation in specific human populations (9), whereas the existence of randomness in the process of genetic variation allows MR to be independent of common confounders and reverse causation (10). In this current study, by identifying single-nucleotide polymorphisms (SNPs) linked to complex traits, a comprehensive bivariate MR analysis was undertaken to identify causal relationships between immunocyte features and GDM. **Figure 1** provides an illustration of this research.

## Materials and methods

### Study design

In this present research, we assessed the causal associations between 731 immunocyte features and GDM using MR analyses. MR employs genetic variants with strong correlations to exposure factors as instrumental variables (IVs), and IVs involved in causal inference have to comply three major assumptions of relevance, independence, and exclusivity (11).

### Sources of data on exposure

The initial immune traits were performed with data from 3,757 Europeans, and the summary data for all traits are available from the publicly available GWAS catalogue (from GCST90001391 to GCST90002121) (12). In order to identify genetic variations occurring in 731 immune cells, GWAS detected approximately 20 million SNPs and 1.6 million indels through high-density genotyping and with reference to Sardinian sequences (13). Specifically, the characterization of immunophenotypes includes four immune trait types with median fluorescence intensities (MFI), relative count (RC), absolute count (AC), and morphological parameter (MP) as well as seven panels with B cell, Treg, myeloid cell, maturation stages, myeloid cell, maturation stages of T cell, monocyte, TBNK, and cDC.

### Sources of data on outcome

The GWAS summary statistics for GDM were derived from finn-bGEST_DIABETES (14). For the study, GWAS were performed on 123,579 Europeans (Ncase = 5,687, Ncontrol = 117,892), and approximately 16 million variants were analyzed after quality control and filtering. All of the original research described above were publicly available, anonymous, and de-identified.

### Screening of relevant IVs

To assure the relevance of causal inferences between immune characteristics and GDM, with reference to previous MR studies, we set the significance threshold of IVs for each immune characteristic at 1 × 10^-5^ (15). Considering the quantity of SNPs obtained, we scaled the significance level for immune cells to 5 × 10^-8^ in reverse extrapolation (16). Since genetic variants with similar genomic locations are more inclined to be co-inherited, in order to assure the independence among genetic tools and remove linkage disequilibrium (LD), we restricted the *r* ^2^ value to 0.001, with a window range of 10,000 kb (*r* ^2 = ^2 × MAF × [1 - MAF] × [β/SD]^2^; MAF: minor allele frequency; β: effect value of SNP on exposure factors) (17). In addition, a strong or weak bias in IVs also leads to weaker correlations with exposure. We have consequently selected the obtained IVs by means of F-statistic (*F* = (*N* - K - 1) × *r* ^2^/[(1 - *r* ^2^) × *K*]; *N*: sample size of GWAS; *K*: number of IVs), when the *F* value is above 10, it is generally thought that there are no significantly weak IVs (18).

### Statistical analysis

We conducted MR analysis with the two-sample MR package (version 0.5.8) in R software (version 4.3.2) and adopted inverse-variance weighted (IVW) as the primary analytical method to estimate the causal effects of genes on traits (19, 20). Statistical approaches such as MR Egger, weighted median, and simple and weighted mode were also utilized to validate the MR results (21, 22). As a widely applied method of MR analysis, IVW is characterized by regressions that ignore the presence of the intercept term and are fitted with the inverse of the outcome variance as weights. MR Egger enables the estimation of bias among causal relationships when there is significant horizontal pleiotropy between SNPs. Even though half of the data were derived from genetic variation in invalid SNPs, the weighted median still yielded a consistent evaluation of causality. Furthermore, leave-one-out method was carried out to remove each SNP one by one, determine the meta impact of the SNPs that remained, and track the modifications in the results following the removal of every SNP (23). If the results changed remarkably after removing a certain SNP, it means that it has a significant effect on the results.

## Results

### Determining the causal role of immunophenotypes in GDM

**Figure 2:**
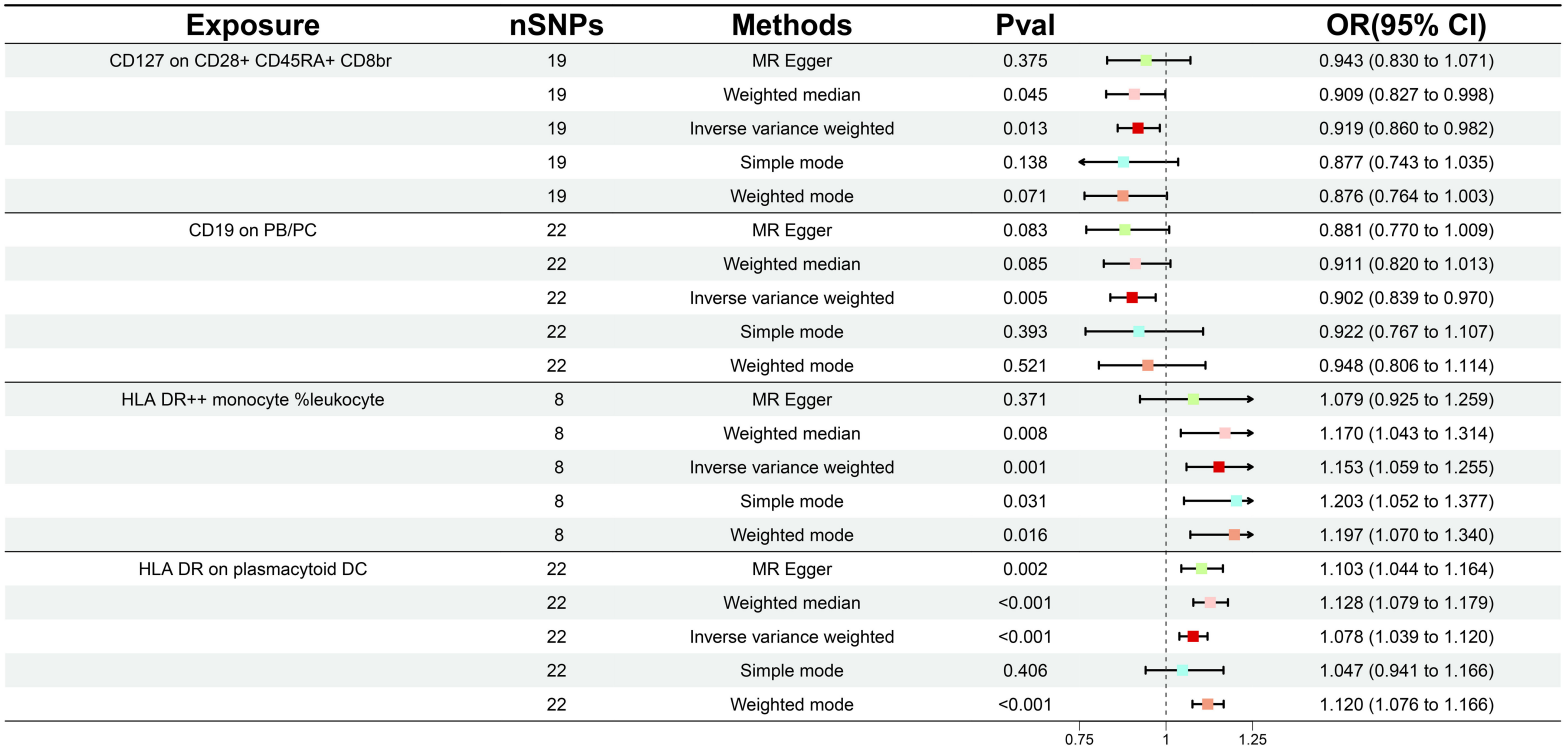
Forest plot illustrating the various ways in which the four immunological features and gestational diabetes mellitus are causally related.

During our investigation, we performed two-sample MR analyses utilizing the IVW method as the principal methodology. After F-statistics as well as an initial significance test, a total of 34 immune cells were detected to exhibit a causal association on GDM, including twelve B cell, seven Treg, four monocyte, four maturation stages of T cell, three TBNK, three cDC, and one myeloid cell panel. With further adjustment for FDR (*P_FDR_* < 0.04), we identified a total of four GDM risk immunophenotypes, which were respectively classified as Treg, B cell, TBNK, and cDC panel (**Supplementary Table S1**). Since then, we have found that certain alterations in the immune milieu influenced the progression of GDM. Concretely, the odds ratio (OR) of *CD127 on CD28+ CD45RA+ CD8br* on GDM risk was estimated to be approximately 0.919 (95% CI: 0.860–0.982, *P* = 0.0125, *P_FDR_* = 0.040) by the IVW method, whereas the MR Egger (95% CI: 0.830–1.071, OR = 0.943, *P* = 0.375) and weighted median (95% CI: 0.827–0.998, OR = 0.909, *P* = 0.045) analyses were consistent with the IVW. Simple mode (95% CI: 0.743–1.035, OR = 0.877, *P* = 0.138) and weighted mode (95% CI: 0.764–1.003, OR = 0.876, *P* = 0.071) also supported the genetic causal inference. The OR of *CD19 on PB/PC* on GDM risk was estimated to be 0.902 (95% CI: 0.839–0.970, *P* = 0.005, *P_FDR_* = 0.038) by the IVW method, whereas the MR Egger (95% CI: 0.770–1.009, OR = 0.881, *P* = 0.083) and weighted median (95% CI: 0.820–1.013, OR = 0.911, *P* = 0.085) analyses were consistent with the IVW. The OR of *HLA DR on plasmacytoid DC* on GDM risk was estimated to be approximately 1.078 (95% CI: 1.039–1.120, *P* = 8.76 × 10^-5^, *P_FDR_* = 0.003) by the IVW method, whereas MR Egger (95% CI 1.044-1.164, OR = 1.103, *P* = 0.002) and weighted median (95% CI 1.079-1.179, OR = 1.128, *P* = 1.07×10^-7^) analyses were consistent with the IVW. The odds ratio of *HLA DR++ monocyte %leukocyte* on GDM risk was estimated to be approximately 1.153 (95% CI 1.059-1.256, *P* = 0.001, *P_FDR_* = 0.018) by the IVW method, whereas the MR Egger (95% CI: 0.924–1.259, OR = 1.079, *P* = 0.371) and weighted median (95% CI: 1.043–1.314, OR = 1.170, *P* = 0.008) analyses were consistent with the IVW (**Figure 2**; **Supplementary Table S2**). Scatter plots and leave-one-out plots also support the stability of the results (**Supplementary Figures S1, 2**).

### Inferring causality of GDM on immunophenotypes

**Figure 3:**
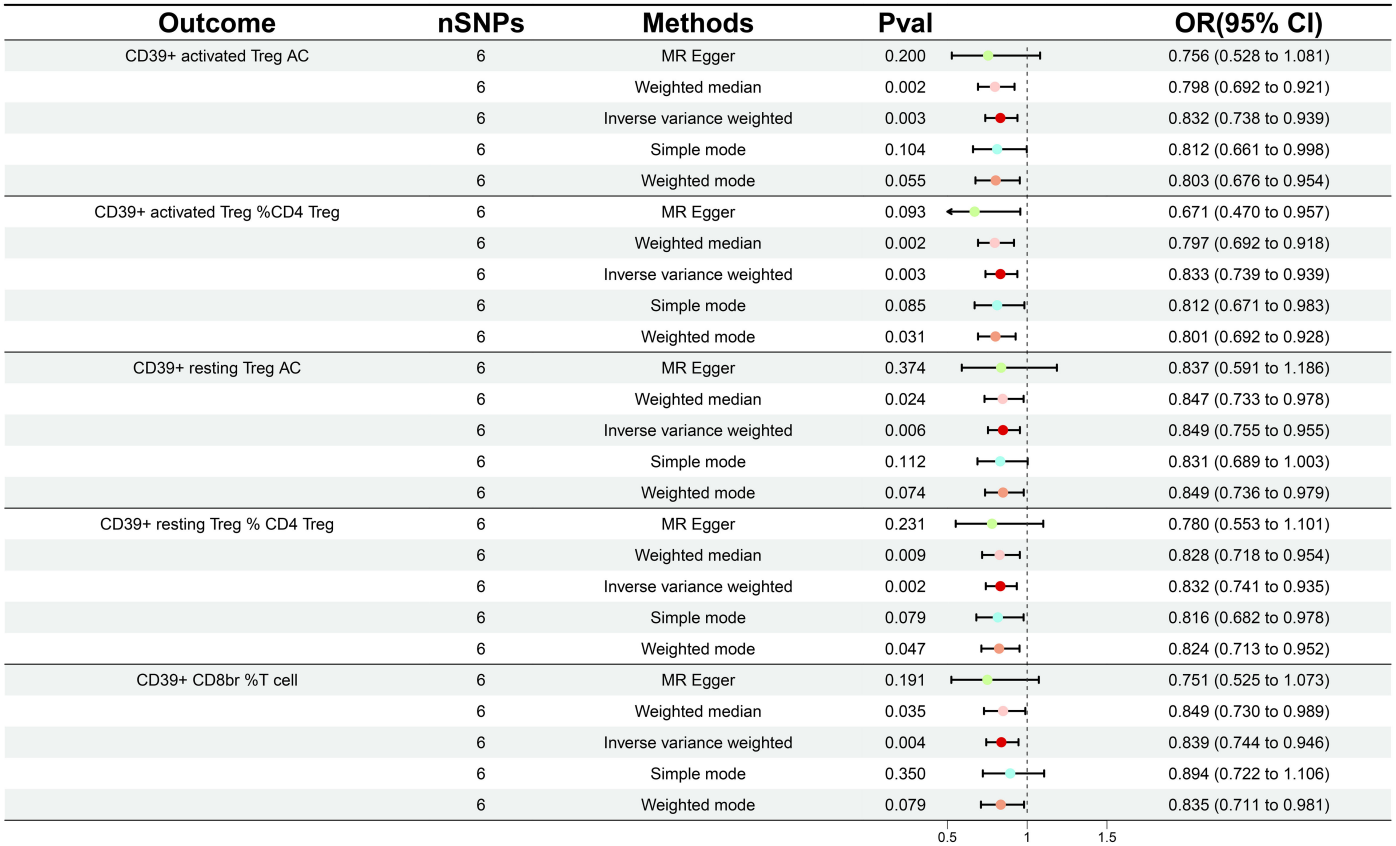
Forest plot illustrating the various ways in which gestational diabetes mellitus and five immunological features are causally related.

In order to ascertain the relationship between the onset and development of GDM with the body’s immunity, we verified the causality of GDM on 36 immune traits. Following GDM, the levels of five immunological features were found to change significantly (**Supplementary Table 3**); all of these traits belonged to the Treg panel and were adjusted for FDR (*P_FDR_* < 0.03). Interestingly, We found that GDM caused the *CD39+ activated Treg %CD4 Treg* (*β* = -0.183, 95% CI: 0.739–0.939, *P* = 0.003, *P_FDR_* = 0.024), *CD39+ activated Treg AC* (*β* = -0.183, 95% CI: 0.738–0.939, *P* = 0.003, *P_FDR_* = 0.021), *CD39+ resting Treg % CD4 Treg* (*β* = -0.183, 95% CI: 0.741–0.935, *P* = 0.002, *P_FDR_* = 0.024), *CD39+ resting Treg AC* (*β* = -0.164, 95% CI: 0.755–0.955, *P* = 0.006, *P_FDR_* = 0.028), and *CD39+ CD8BR %T cell* (*β* = -0.176, 95% CI: 0.744–0.946, *P* = 0.004, *P_FDR_* = 0.025) levels to show a similar decrease (**Figure 3**; **Supplementary Table S4**). The results from other MR methods and sensitivity analyses demonstrate the robustness of the observed causal associations (**Supplementary Figures S3, 4**).

## Discussion

GWAS studies have revealed associations between diseases and genetic variation, etc. Benefitting from a large, publicly available genomic data, our study attempted to offer genetic evidence for the causal link between GDM and multiple immune phenotypes. In this paper, we proved that four immune phenotypes have a significant causal association for GDM (*P_FDR_* < 0.04), while GDM has the same relationship for five other immune phenotypes (*P_FDR_* < 0.03).

As a globally prevalent obstetric disorder, GDM has been shown to be associated with many adverse maternal and fetal pregnancy outcomes. Abnormal maternal immune adaptation is key to the low-grade inflammation associated with the diagnosis of GDM, while immune cell infiltration of visceral adipose tissue causes the pathological dysregulation of insulin signaling and contributes to insulin resistance. Specifically, CD39 is located on the surface of trophoblast cells in the normal human placenta and regulates ATP-dependent trophoblast function, which is critical for immune tolerance and the maintenance of a normal pregnancy (24). Several studies have shown that measuring CD19+ subpopulations can help predict the pregnancy outcome in women, with a trend towards lower peripheral CD19+ B cells in women who miscarry compared to those who subsequently give birth (25). The frequency of CD19(+) CD5(+) cells was also significantly increased in the peripheral blood of patients with pre-eclampsia compared to normal pregnant women (26). In addition, the maternal humoral response to fetal anti-HLA-DR immunoglobulin antibodies may influence the development of pregnancy-induced hypertension (27).

Our results demonstrate that the elevated levels of *HLA DR on plasmacytoid DC* and *HLA DR++ monocyte %leukocyte* increase the risk of GDM. As a specialized antigen-presenting cell, dendritic cells (DCs) regulate the immune response and bridge the gap between innate and adaptive immunity (28). DCs play crucial roles in the growth and development of embryos and fetuses in the mother’s womb; dysregulation of the DC subpopulations appears to be linked to adverse pregnancy outcomes (29). HLA-DR is the most common MHC class II molecule on the surface of antigen-presenting cells. Specially, the expression of HLA-DR on the surface of DCs increases the abundance of protein complexes and is accompanied by the production of co-stimulatory molecules and cytokines (30). Moreover, recent studies have demonstrated that the level of HLA-DR is an indicator of monocyte immunocompetence, which not only assists in antigen presentation but also strengthens TLR-2-mediated signaling, cell proliferation, and maturation (31).

On the contrary, the growing levels of *CD127 on CD28+ CD45RA+ CD8br* and *CD19 on PB/PC* decrease the risk of GDM. The reduced activity of inhibitory Treg isoforms in GDM was pointed out to be associated with the upregulation of pro-inflammatory factor concentrations which include IL-6 and TNF-alpha (32). Another study showed that the percentage of circulating Treg subpopulation cells defined by *CD3+CD4+CD25 bright/dim CD127* expression was reduced in GDM pregnancies compared with glucose-tolerant pregnancies (33). CD19 is a transmembrane protein on the surface of B cells, which is tightly connected with B cell activation, signaling, and growth regulation. For IgG4-related diseases, the peripheral blood was significantly enriched in B cell populations, including *CD19+ CD24-CD38hi PB/PC*. After glucocorticoid administration, the levels of these cells declined, accompanied by an improvement in clinical symptoms (34). In summary, our research indicates that immune cells have a significant role in GDM’s early diagnosis, therapeutic monitoring, disease activity assessment, and adaptive therapies.

On the other hand, we also revealed that the percentage of various immunological phenotypes was altered as a result of GDM. Strikingly, we found that GDM commonly lowered the abundance of *CD39+ activated Treg %CD4 Treg*, *CD39+ activated Treg AC, CD39+ resting Treg % CD4 Treg*, *CD39+ resting Treg AC*, and *CD39+ CD8BR %T cell*. Extracellular ATP is an effective proinflammatory factor *in vivo*, and its hydrolysis is important for its immunosuppressive function. As an extracellular ectonucleotidase, CD39 has been implicated as a major marker of FOXP3+ Treg and cleaves ATP to form AMP in the rate-limiting step. It is notable that the percentage of D39+ Treg cells was significantly decreased in type 2 diabetes patients as compared to the controls. *In vivo* experiments have shown that CD39-deficient mice exhibit impaired glucose tolerance in an oral glucose tolerance test (35). The supplementation of soluble CD39 to pre-diabetic NOD mice reduces the extent of extracellular ATP, inhibits the multiplication of CD4+ T cells, and delays the further progression of diabetes (36). It reminds us that individualized treatment for CD39 is probably a promising option for pregnant women.

Apparently, our study offers a foundation for delineating the intricate causal association between immune cells and GDM. However, there are still several limitations in our work. Firstly, although we performed MR analyses with a large-scale GWAS cohort and avoided potential confounders or reverse causation, genetic heterogeneity among different human populations still attenuates the credibility and validity of the GWAS results. Secondly, when examining the association of immune cells with GDM, a more relaxed threshold was chosen to ensure accurate data on SNPs. Even with the FDR test applied, this may still lead to a minor bias in the results. Finally, for GDM, our study was unable to further probe specific traits (for example—age, weight, and hormone levels) in the group of pregnant women.

## Conclusions

In summary, we emphasized the causal relationship between a number of immune phenotypes and GDM through a full bidirectional MR analysis. To our knowledge, this is the first MR analysis carried out between immune phenotypes and GDM, providing novel insights into understanding the delicate balance between maternal immune mediators and GDM. GDM is a complicated and dynamic condition, and the pathophysiological mechanisms are not fully clarified. This research enables researchers to better explain the physiological mechanisms, with a view to filtering and monitoring high-risk groups for GDM, contributing to the early intervention and the development of new treatments of GDM.

## Data availability statement

The datasets presented in this study can be found in online repositories. The names of the repository/repositories and accession number(s) can be found in the article/**Supplementary Material**.

## Author contributions

ZJ: Conceptualization, Data curation, Investigation, Visualization, Writing – original draft. CZ: Conceptualization, Data curation, Visualization, Writing – original draft. JY: Supervision, Validation, Writing – review & editing. QH: Validation, Visualization, Writing – review & editing. XZ: Validation, Visualization, Writing – review & editing. DY: Validation, Visualization, Writing – review & editing. NX: Formal Analysis, Funding acquisition, Project administration, Supervision, Writing – review & editing. JC: Formal Analysis, Funding acquisition, Project administration, Supervision, Writing – review & editing.
