# Supplementary figures and images for: Is there a causal association between gestational diabetes mellitus and immune mediators? A bidirectional Mendelian randomization analysis
Source: Front Endocrinol (Lausanne). 2024 Apr 19;15:1358144. doi: 10.3389/fendo.2024.1358144 (PMC11066251; doi:10.3389/fendo.2024.1358144)

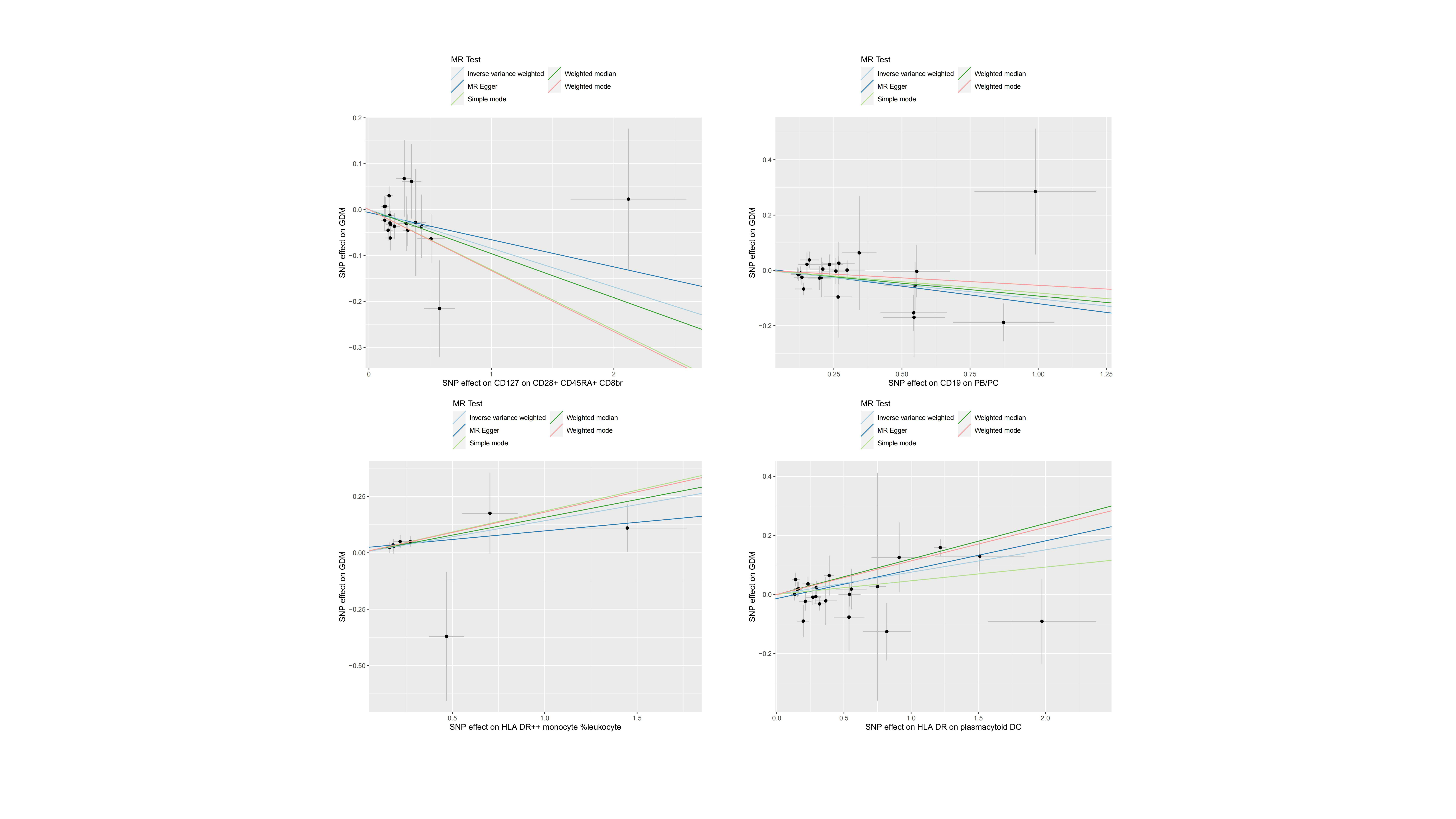

Supplement: Supplementary file 1 [file Image_1.tif]

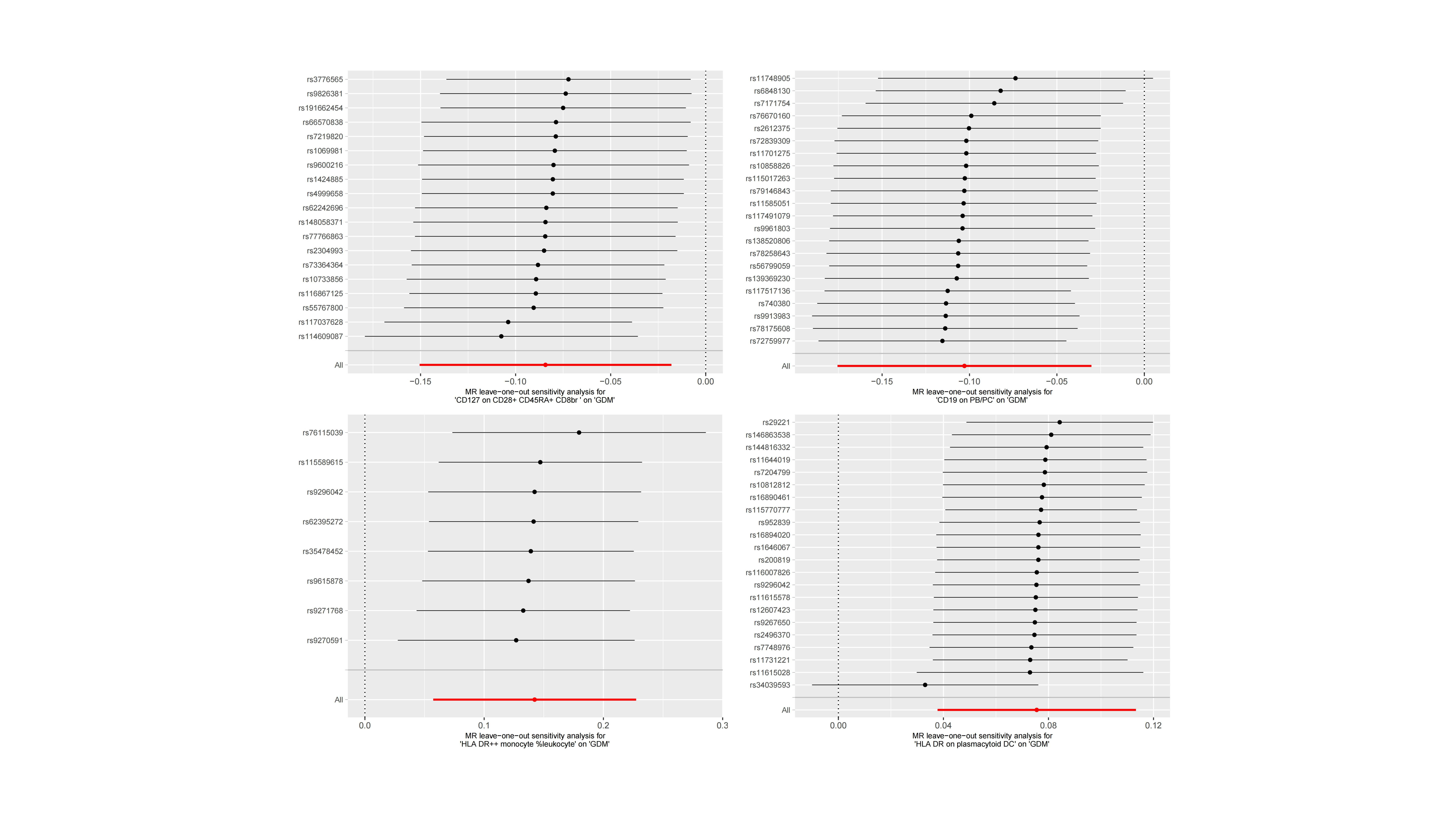

Supplement: Supplementary file 2 [file Image_2.tif]

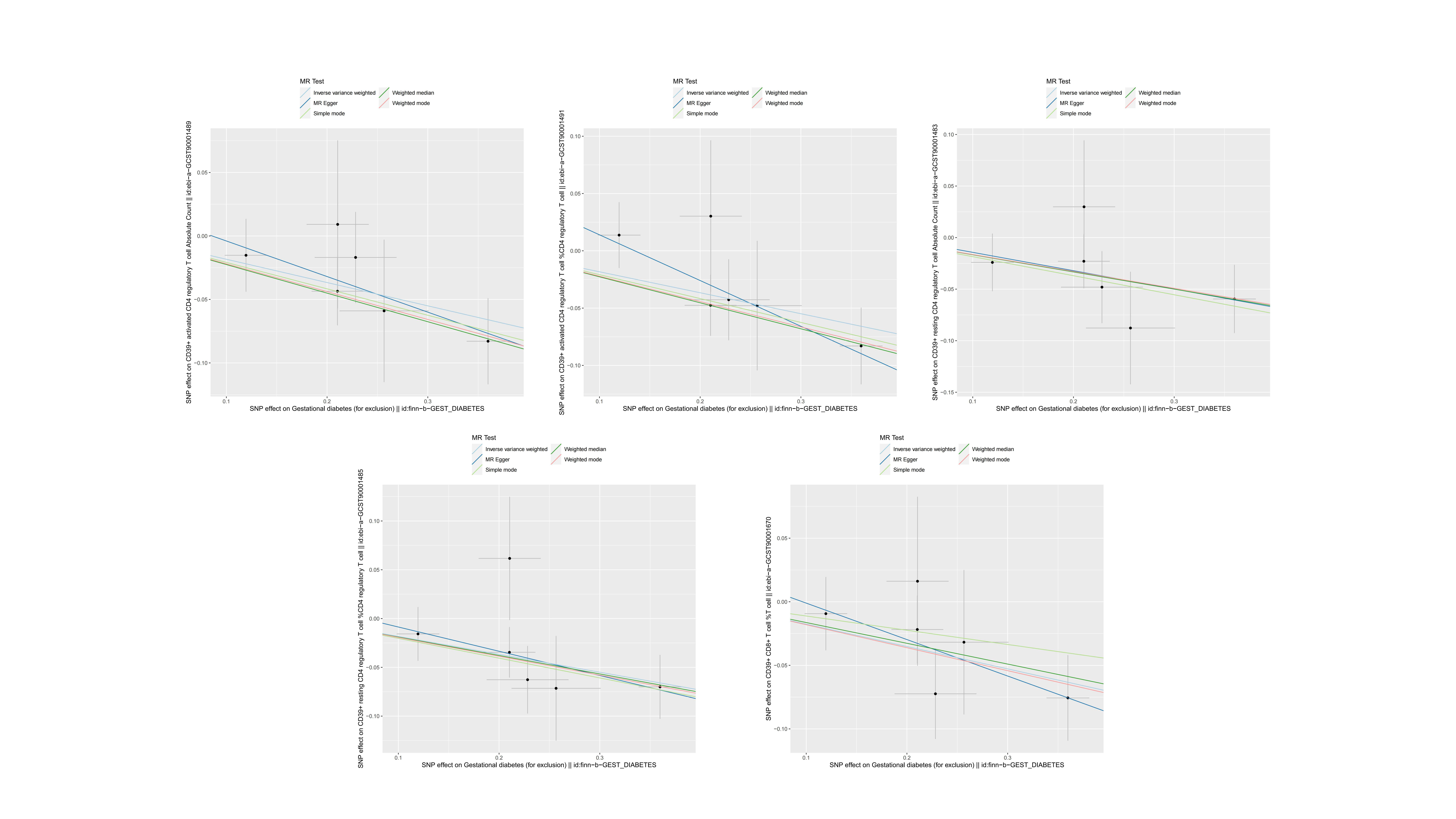

Supplement: Supplementary file 3 [file Image_3.tif]

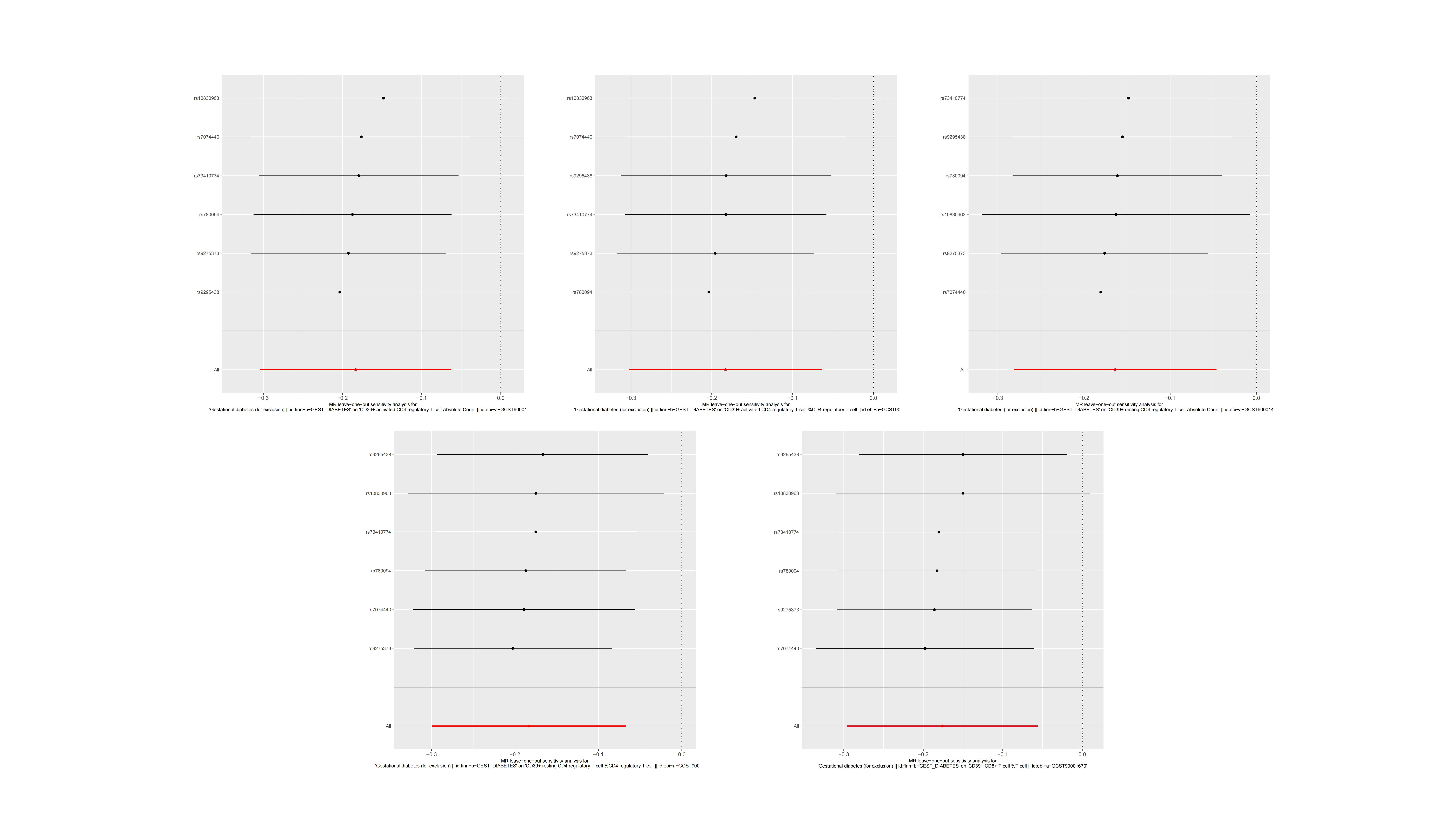

Supplement: Supplementary file 4 [file Image_4.tif]
